# Postexpositionsprophylaxe nach Biss einer Breitflügelfledermaus mit Nachweis des Europäischen Fledermaus-Lyssavirus 1 (EBLV-1)

**DOI:** 10.1007/s00108-023-01638-3

**Published:** 2023-12-15

**Authors:** Jannik Fasse, Henning Trawinski, Michael Hardt, Christoph Lübbert

**Affiliations:** 1 Bereich Infektiologie und Tropenmedizin, Medizinische Klinik I (Hämatologie, Zelltherapie, Hämostaseologie und Infektiologie), Universitätsklinikum Leipzig, Liebigstraße 20, Haus 4, 04103 Leipzig, Deutschland; 2 Interdisziplinäres Zentrum für Infektionsmedizin (ZINF), Universitätsklinikum Leipzig, Leipzig, Deutschland; 3 Standort Leipzig, Landesuntersuchungsanstalt für das Gesundheits- und Veterinärwesen Sachsen (LUA), Leipzig, Deutschland; 4 Klinik für Infektiologie und Tropenmedizin, Klinikum St. Georg gGmbH Leipzig, Leipzig, Deutschland

**Keywords:** Tollwut, Postexpositionsprophylaxe, Bissverletzung, Impfung, Europäisches Fledermaus-Lyssavirus 1, Rabies, Postexposure prophylaxis, Bite injury, Vaccination, European bat lyssavirus 1

## Abstract

Deutschland gilt infolge intensiver Impf- und Überwachungsbemühen seit 2008 als frei von terrestrischer Tollwut. Reservoire der Lyssaviren EBLV‑1 und EBLV‑2 persistieren jedoch weiter in Fledermauskolonien und stellen somit ein potenzielles Infektionsrisiko dar. Wir berichten von einer Patientin, die einen Fledermausbiss im städtischen Umfeld erlitt. Bei der euthanasierten Fledermaus konnte das Europäische Fledermaus-Lyssavirus 1 (EBLV-1) nachgewiesen werden. Wir führten eine aktive und passive Postexpositionsprophylaxe (PEP) durch. Dieses Fallbeispiel illustriert die anhaltende Tollwutinfektionsgefahr durch enge Fledermauskontakte in Deutschland und soll erstbehandelnde Ärzte dafür sensibilisieren, entsprechende Expositionsereignisse ernst zu nehmen und eine regelrechte PEP einschließlich Applikation von Tollwutimmunglobulin durchzuführen.

## Abstract (en)

Germany has been considered free of terrestrial rabies since 2008 as a result of intensive vaccination and surveillance efforts but reservoirs of the lyssaviruses EBLV‑1 and EBLV‑2 persist in bat colonies and thus pose a potential risk of infection. We report on a patient who suffered a bat bite in an urban setting in which European bat lyssavirus 1 (EBLV-1) was detected in the euthanized bat. We performed active and passive postexposure prophylaxis (PEP). This case study illustrates the ongoing risk of rabies infection due to close bat contacts in Germany and is intended to sensitize primary care physicians to take such exposure events seriously and to perform a regular PEP including administration of rabies immunoglobulin.

## Anamnese

Eine 59-jährige Frau aus dem Raum Leipzig stellte sich spontan in unserer infektiologischen Ambulanz vor, nachdem sie am Vorabend von einer Breitflügelfledermaus (*Eptesicus serotinus*) in den kleinen Finger der linken Hand gebissen worden sei. Die Fledermaus lag wegen einer Flügelverletzung immobil am Boden und wurde von der Patientin hochgehoben. Die verletzte Fledermaus wurde von einem ehrenamtlichen Mitarbeiter der Ortsgruppe des Naturschutzbundes Deutschland (NABU) zur tierärztlichen Versorgung gebracht, nachdem dieser von der Patientin telefonisch informiert worden war.

## Befund

Der Hautmantel im Bereich des gebissenen Fingers war inspektorisch intakt. Die letzte Tetanusauffrischungsimpfung erfolgte 2019. Eine Tollwutimmunisierung wurde bei der Patientin bisher nicht durchgeführt.

## Diagnose

Mögliche Tollwutexposition nach Fledermausbissverletzung im Bereich der linken Hand

## Weiterer Verlauf

**Figure 1:**
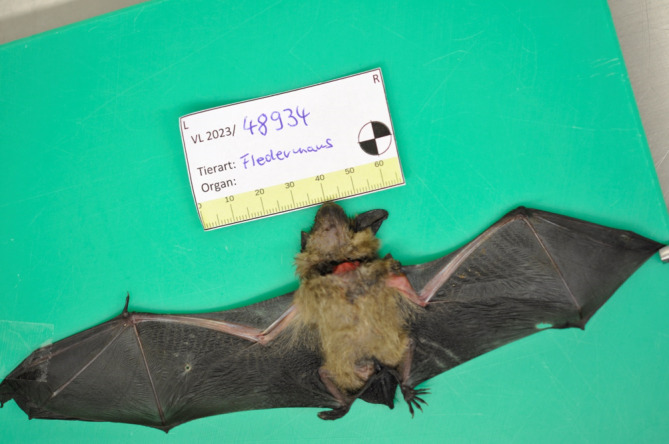

Die Fledermaus wurde aufgrund des Flügelbruchs tierärztlich euthanasiert und der zuständigen Landesuntersuchungsanstalt für das Gesundheits- und Veterinärwesen Sachsen (LUA) zur Autopsie übergeben (Abb. 1 und 2). Dort konnte zunächst in der Immunfluoreszenzmikroskopie (Abb. 3), anschließend auch mithilfe einer spezifischen PCR-Untersuchung das Europäische Fledermaus-Lyssavirus 1 (EBLV-1) im zentralen Nervensystem der Fledermaus nachgewiesen werden. Die virologische Untersuchung auf Tollwutviren mittels Anzucht auf neuronalen NA-Zellen war ebenfalls positiv.

**Figure 2:**
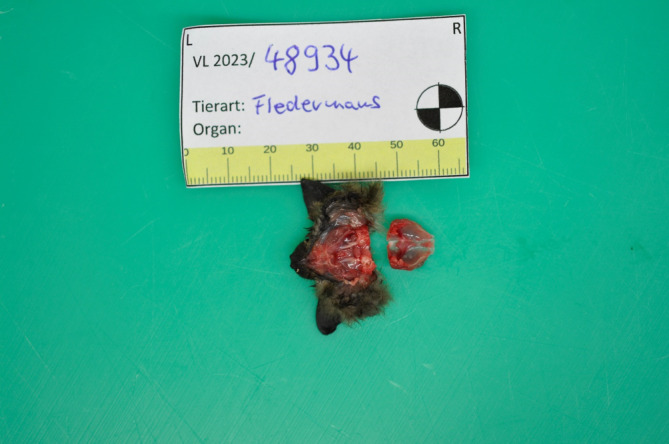

**Figure 3:**
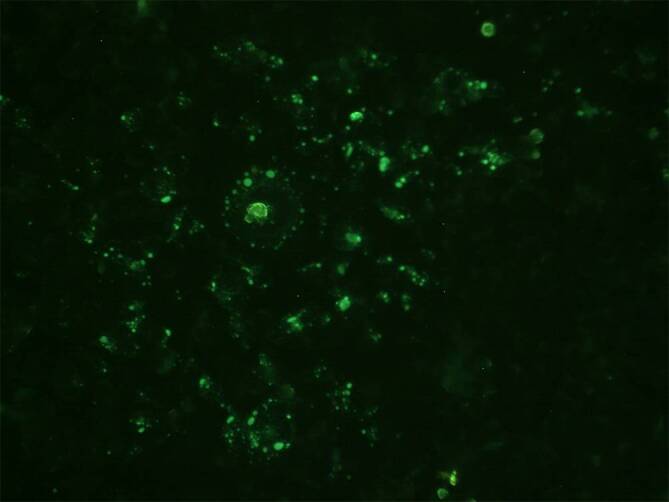

## Therapie und Nachbeobachtung

Bei nicht vorhandener Grundimmunisierung führten wir gemäß Empfehlung der Ständigen Impfkommission beim Robert Koch-Institut (STIKO) sowohl eine passive PEP mit sofortiger lokaler Applikation von Tollwutimmunglobulin (TIG) in gewichtsadaptierter Dosierung (20 IE/kgKG) als auch eine aktive Impfung mit einem zellkulturbasierten Tollwutimpfstoff nach dem Essen-Schema (Tag 0, 3, 7, 14, 28) durch [1]. Die Erstgabe erfolgte ca. 16 h nach der Bissverletzung. Die Immunglobulingabe sowie die aktiven Impfungen wurden gut vertragen. Die klinische Nachbeobachtung der Patientin blieb unauffällig.

## Diskussion

Auch wenn Deutschland als frei von terrestrischer Tollwut gilt, besteht durch Fledermausbisse, -kratzer oder -schleimhautkontakte weiter ein relevantes Tollwutinfektionsrisiko [2, 3]. Nach heutigem Wissensstand wird die Fledermaustollwut in Europa durch 3 Virusvarianten, die Europäischen Fledermaus-Lyssaviren 1 und 2 (EBLV‑1 und -2) und das Bokeloh-Fledermaus-Tollwutvirus (BBLV), verursacht [2, 3]. Diese Viren sind nicht mit dem Erreger der klassischen Fuchstollwut (Rabiesvirus [RABV]) identisch.

Auch wenn in Deutschland das Tollwutimmunglobulin (TIG) breit verfügbar ist, kommt es bei der PEP-Durchführung häufiger zu Fehlanwendungen [4], sodass Expositionen prinzipiell vermieden werden sollten und bei der PEP-Durchführung auf qualifizierte Expertise im eigenen Haus oder extern (Tollwutberatungsstellen) zurückgegriffen werden sollte [3]. Empfehlungen zum Umgang mit gefundenen Fledermäusen gibt beispielsweise der Naturschutzbund Deutschland (NABU; [5]). Keineswegs sollte diese Betrachtung zu einer Stigmatisierung der unter Naturschutz stehenden Fledermausarten führen.

Die Tollwut-PEP gilt als hochwirksam. Dennoch wurde über sporadische Durchbruchsinfektionen (d. h. Tollwut bei Personen, die eine PEP begonnen haben) berichtet [3]. Ein aktueller systematischer Review der Jahre 1980 bis 2022 identifizierte 122 publizierte Durchbruchsinfektionen weltweit [6]. Die mittlere Zeit von der Exposition bis zum Auftreten von Symptomen betrug 20 Tage. 77 % der Patienten erhielten innerhalb von 2 Tagen nach Exposition ihre PEP. Schwere Wunden (definiert als solche mit mehreren Wundstellen oder Bisswunden an Kopf, Gesicht oder Hals) waren häufig (69 % der Fälle). In 56 % der Fälle wurden Protokollabweichungen von den üblichen PEP-Standards festgestellt. Weitere mögliche Ursachen für Durchbruchsinfektionen waren Fehler bei der Verabreichung von TIG, Verzögerungen bei der Inanspruchnahme von medizinischer Versorgung sowie schwere Komorbiditäten oder Immunsuppression (insbesondere Kortikosteroidtherapie; [6]). Bewertungen der Integrität der Kühlkette und Wirksamkeitstests der verwendeten PEP-Vakzine wurden nur selten durchgeführt (7 % der Fälle), und in keinem dieser Fälle ließ sich eine Ursache für die Durchbruchsinfektion finden [6].

Im Jahr 2023 wurde aus Minnesota (USA) bei einem 84-jährigen Patienten die erste Tollwutdurchbruchsinfektion in der westlichen Hemisphäre bei Verwendung eines modernen zellkulturbasierten Tollwutimpfstoffs publiziert [7]. Die wahrscheinlichste Erklärung für diese Durchbruchsinfektion ist ein durch den Wirt vermitteltes Versagen des Primärimpfstoffs, welches von den Autoren auf das fortgeschrittene Alter und die Begleiterkrankungen (u. a. ein erst bei der Autopsie festgestelltes Prostatakarzinom) zurückgeführt wurde. Kliniker sollten daher 14 Tage nach Abschluss der PEP eine Titermessung der neutralisierenden Tollwutantikörper im Blut des Patienten in Erwägung ziehen, wenn der Verdacht auf eine relevante Immunschwäche besteht [6].

## Fazit für die Praxis

Dieses Fallbeispiel soll die Tollwutinfektionsgefahr durch Fledermausbisse unterstreichen und behandelnde Erstversorger dazu anhalten, potenzielle Expositionen ernst zu nehmen. Fledermausbisswunden sind aufgrund der feinen Zähne nicht immer ersichtlich und eine PEP-Indikation dementsprechend großzügig zu stellen. Personen mit regelmäßigem Kontakt zu Fledermäusen sollten grundimmunisiert werden und regelmäßige Auffrischungsimpfungen gemäß den Empfehlungen der STIKO erhalten.

## Abkürzungen

EBLV‑1: „European bat lyssa virus 1“
LUA: Landesuntersuchungsanstalt für das Gesundheits- und Veterinärwesen Sachsen
PEP: Postexpositionsprophylaxe
TIG: Tollwutimmunglobulin
